# SR-BI: Linking Cholesterol and Lipoprotein Metabolism with Breast and Prostate Cancer

**DOI:** 10.3389/fphar.2016.00338

**Published:** 2016-10-07

**Authors:** Jorge L. Gutierrez-Pajares, Céline Ben Hassen, Stéphan Chevalier, Philippe G. Frank

**Affiliations:** Université François Rabelais de Tours, Faculté de Médecine-INSERM UMR1069 “Nutrition, Croissance et Cancer”Tours, France

**Keywords:** SR-BI, cholesterol, lipoprotein, HDL cholesterol, breast cancer

## Abstract

Studies have demonstrated the significant role of cholesterol and lipoprotein metabolism in the progression of cancer. The *SCARB1* gene encodes the scavenger receptor class B type I (SR-BI), which is an 82-kDa glycoprotein with two transmembrane domains separated by a large extracellular loop. SR-BI plays an important role in the regulation of cholesterol exchange between cells and high-density lipoproteins. Accordingly, hepatic SR-BI has been shown to play an essential role in the regulation of the reverse cholesterol transport pathway, which promotes the removal and excretion of excess body cholesterol. In the context of atherosclerosis, SR-BI has been implicated in the regulation of intracellular signaling, lipid accumulation, foam cell formation, and cellular apoptosis. Furthermore, since lipid metabolism is a relevant target for cancer treatment, recent studies have focused on examining the role of SR-BI in this pathology. While signaling pathways have initially been explored in non-tumoral cells, studies with cancer cells have now demonstrated SR-BI's function in tumor progression. In this review, we will discuss the role of SR-BI during tumor development and malignant progression. In addition, we will provide insights into the transcriptional and post-transcriptional regulation of the *SCARB1* gene. Overall, studying the role of SR-BI in tumor development and progression should allow us to gain useful information for the development of new therapeutic strategies.

## Introduction

In human, cholesterol is transported by lipoproteins [i.e., low-density lipoproteins (LDL) and high-density lipoproteins (HDL)], which permit its transfer to tissues. Cholesterol elimination from the body is performed via the reverse cholesterol transport pathway, which allows the transfer of peripheral cholesterol to HDL and eventually its transfer to the liver for excretion. Interestingly, solid tumors have been shown to accumulate greater amounts of cholesterol when compared to the host healthy tissues (Freeman and Solomon, 2004; Krycer and Brown, 2013; de Gonzalo-Calvo et al., 2015). Intratumor accumulation of cholesterol appears to be a consequence of both *de novo* synthesis and lipoprotein-mediated uptake (de Gonzalo-Calvo et al., 2015; Murai, 2015). In this mini-review, we will focus on the role of the HDL receptor, the scavenger receptor class B type I (SR-BI) in the regulation of cholesterol and lipoprotein metabolism in the context of cancer. Although SR-BI's contributions to reverse cholesterol transport in cardiovascular diseases have been extensively studied, recent evidence has suggested that cholesterol and its metabolites may play a critical role in cancer progression (Danilo and Frank, 2012; Silvente-Poirot and Poirot, 2012; Simko and Ginter, 2014; Kuzu et al., 2016).

## SR-BI: initial characterizations

SR-BI is a member of the Class B family of Scavenger Receptor proteins, which also include CD36 Antigen-like2 (LIMPII) and CD36 (Calvo et al., 1995). These three glycoproteins share a common structure: two transmembrane domains associated with two intracellular N- and C-termini and an extracellular glycosylated central domain. Initially, human SR-BI was termed CD36 and LIMPII Analogous-1 (CLA-1; Calvo and Vega, 1993) and was found to be highly expressed in adrenal glands (Liu et al., 1997), liver and steroidogenic tissues (Calvo et al., 1997). SR-BI is a receptor for HDL, and it promotes selective HDL-cholesteryl ester (HDL-CE) uptake by cells without particle uptake (Silver et al., 2000; Trigatti et al., 2000). Additionally, studies have shown that SR-BI can also promote the elimination of excess body cholesterol via billiary cholesterol secretion (Harder et al., 2007; Wiersma et al., 2009a,b).

## *SCARB1* gene localization, splice variants, and protein domains

The gene encoding SR-BI has been designated *SCARB1*. Human *SCARB1* is located on chromosome 12 at q24.31 and comprises 13 exons and 12 introns that span over 86 kb. Due to alternative splicing sites, several mRNA variants of *SCARB1* have been identified (Webb et al., 1997, 1998). Interestingly, a short variant containing only the last 2 exons of *SCARB1* has been detected by next-generation sequencing in non-malignant adrenal glands and livers at relatively high levels (Carithers et al., 2015). Nevertheless, no experimental data has been reported on the physiological significance of this finding.

The predicted molecular weight of SR-BI is 56.9 kDa, but it is frequently detected as an 82 kDa protein after SDS-PAGE migration due to post-translational glycosylation (Acton et al., 1994; Babitt et al., 1997). Using SR-BI aminoacid sequence Q8WTV0-2 (UniProt, 2015), the following main domains of SR-BI can be identified: Cytoplasmic N-terminal domain (residues 1–11), transmembrane domain #1 (residues 12–32), extracellular domain (residues 33–440), transmembrane domain #2 (residues 441–461), and cytoplasmic C-terminal domain (residues 462–509). According to the UniProt website (accessed July 2016, UniProt, 2015), five protein variants may be produced by alternative splicing of human *SCARB1* mRNA: isoform 3 (Q8WTV0-1), the canonical sequence, represents the longest variant with 552 residues; isoform 1 (Q8WTV0-2), the first isoform identified and named SR-BI, with 509 residues; isoform 2 (Q8WTV0-3; aka, SR-BII), with 409 residues; isoform 4 (Q8WTV0-2; aka, SR-BIII), with 474 residues; and isoform 5 (Q8WTV0-5), with 506 residues. Isoforms 1, 2, and 4 share a common C-terminal sequence (468–552 aa) that includes the VLQEAKL sequence required to bind the PDZ domain-containing protein (PDZK1), which has also been described in the mouse sequence (Silver, 2002; Kocher et al., 2010). The physiological relevance of these variants is not evident but SR-BII has been shown to display reduced selective cholesteryl ester uptake efficiency from HDL (Webb et al., 1998). SR-BI isoform distribution may also be altered in certain types of cancer (Arenas et al., 2004), compared to the corresponding healthy tissues, and it is possible that different SR-BI isoforms may have different ability to promote cholesterol entry or efflux. Therefore, these isoforms may differently regulate cholesterol homeostasis and/or signaling pathways involved in tumor progression.

## Regulations of *SCARB1* transcription and SR-BI protein levels

Steroidogenic tissues (Cao et al., 1997; Azhar et al., 2003) and the liver (Malerød et al., 2002; Huby et al., 2006) present the highest levels of *SCARB1* transcript. Accordingly, a strong control over transcription of this gene has been recognized. mRNA transcription of *SCARB1* is finely regulated in cells that perform steroidogenesis, such as the ovaries (Li et al., 1998) and adrenal glands (Imachi et al., 1999). It has been shown that the adrenocorticotropic hormone (ACTH), via cAMP, can stimulate SR-BI expression in human adrenocortical cell lines and promotes the secretion of aldosterone and cortisol (Imachi et al., 1999). However, no canonical cAMP responsive element (CRE) has been found in the promoter of *SCARB1* (Towns and Menon, 2005). This finding suggests that activation via CREB may occur via non-canonical CRE elements or cAMP regulates the activity of other transcription factors. Several activators of *SCARB1* transcription have been identified in a variety of cell types. Activation by Peroxisome proliferator-activated receptors (PPARα and PPARγ) promotes transcription of *SCARB1* in monocytes and macrophages (Chinetti et al., 2000). Similarly, activation of the Mek1/2/PPARα pathway has been demonstrated to up-regulate SR-BI (Wood et al., 2011). SREBP-2, which is a key regulator of cholesterol metabolism, binds *SCARB1* promoter to induce *SCARB1* mRNA up-regulation (Tréguier et al., 2004). LXRα/RXR and LXRβ/RXR have also been shown to induce *SCARB1* transcription in human and murine hepatoma cell lines and in 3T3-L1 preadipocytes independently of SREBP-1 (Malerød et al., 2002). In agreement with these findings, the LXR agonist T0901317 but not 22-(R)-hydroxycholesterol has been shown to induce an up-regulation of *SCARB1* mRNA in endothelial cells (Norata et al., 2005). Finally, 17β-estradiol has also been shown to transcriptionally up-regulate SR-BI via PKC activation in vascular endothelial cells (Fukata et al., 2014). Taken together, these data show that regulation of *SCARB1* transcription is strongly controlled by signaling pathways involved in cellular lipid/cholesterol homestasis. Therefore, it is not surprising to observe an upregulation of SR-BI in cancer cells (Table 1), which often display increased cellular cholesterol levels.

**Table 1:** **Expression of SR-BI in cancer tissues or cell lines and its association with malignant features**.

**Type of cancer**	**Type of sample**	**Expression levels (compared to non-malignant tissue or cells)**	**Associated with**	**References**
Adrenocortical carcinomas	Tissue	Decreased	ND	Liu et al., 1997
Testicular seminoma	Tissue	Decreased	ND	Arenas et al., 2004
Leydig cell tumor	Tissue	Increased	ND	Liu et al., 1997
Breast	Tissue	Increased	ND	Cao et al., 2004
Breast	Tissue	Increased	More aggressive tumor type	Yuan et al., 2016
Prostate	Tissue	Increased	Increased malignancy and androgen-independent growth	Schorghofer et al., 2015
Nasopharyngeal carcinoma	Tissue	Increased	ND	Zheng et al., 2013
Nasopharyngeal carcinoma	Cell line	Increased	Enhanced cell migration but not proliferation	Zheng et al., 2013
Breast	Cell line	NA	Enhanced proliferation	Cao et al., 2004
Breast	Cell line	NA	Enhanced proliferation and migration, and tumor growth in xenograft tumor model	Danilo et al., 2013
Prostate	Cell line	NA	PSA production and cell viability	Twiddy et al., 2012
Breast	PyMTTg mouse model	Increased	Tumor growth	Llaverias et al., 2011
Prostate	TRAMP mouse model	Increased	Tumor growth	Llaverias et al., 2010

ND, not determined; NA, not applicable.

Furthermore, recent studies have also shown that the proto-oncogene c-Myc may play a role in the regulation of *SCARB1* transcription (Pello et al., 2012). Moreover, *in silico* nucleotide analysis of *SCARB1* promoter (nucleotide −499 to 100, LASAGNA Search 2.0) shows potential binding sites for c-Myc, Stat proteins, PPARγ, p53, and NF-κB. Given the role of transcription factors such as c-Myc, p53, Stat in cancer, additional studies should be designed to better understand the role of these transcription factors in the regulation of SR-BI expression in cancer.

In addition to transcription factors regulating gene expression, mRNA levels of *SCARB1* can also be regulated by microRNAs (miRNAs), which can bind the 3′-UTR of mRNA. Studies have shown that miR-125a and miR-455 can reduce both mRNA and protein levels of SR-BI in steroidogenic cells (Hu et al., 2012). Similarly, miR-185, -96, and -223 have been shown to decrease mRNA levels of *SCARB1* in HepG2 cells (Wang et al., 2013). Finally, miR-192 was found to directly target *SCARB1* mRNA in adipocytes (Mysore et al., 2016). Although no data on the role of these miRNAs in the context of *SCARB1* and cancer is available, recent studies have shown that levels of miR-125a (Guo et al., 2009; He et al., 2015; Tong et al., 2015; Lee et al., 2016), miR-455 (Chai et al., 2015; Li et al., 2016), miR-185 (Qu et al., 2013; Fu et al., 2014; Tang et al., 2014; Li et al., 2015; Zhang et al., 2015), and miR-192 (Feng et al., 2011; Geng et al., 2014; Sun et al., 2016) are down-regulated in several types of cancer. These findings suggest that *SCARB1* post-transcriptional regulation may be altered to promote SR-BI expression in cancer cells.

A post-translational regulation of SR-BI has also been described. In this case, PDZK1 has been shown to physically interact with SR-BI via a 7-residue domain present in the C-terminal domain (Silver, 2002; Kocher et al., 2010). This interaction was demonstrated to be essential for SR-BI post-transcriptional stabilization in a tissue-dependent manner (Kocher et al., 2003) and localization to the plasma membrane of hepatocytes (Silver, 2002; Kocher et al., 2003) but not relevant for its selective HDL-CE uptake function (Silver, 2002). Importantly, phosphorylation of serine 509 of PDZK1 is crucial for SR-BI stabilization (Nakamura et al., 2005). Despite these findings, the role of PDZK1 in SR-BI post-translational regulation does not appear to be required in all cell types. In fact, absence of PDZK1 does not affect SR-BI levels in murine steroidogenic tissues (Kocher et al., 2003) and human endothelial cells (Kimura et al., 2006). Remarkably, recent studies have demonstrated that PDZK1 expression is correlated with Akt activation in the MCF-7 breast cancer cell line (Kim et al., 2014). This observation implies a positive feedback regulation between PI3K/AKT and SR-BI since PI3K is activated by SR-BI (Kimura et al., 2006). Alternatively, SR-BI protein levels may be regulated by Mek1/2, which prevents SR-BI proteosomal degradation in hepatocytes (Wood et al., 2011). Taken together, these studies highlight the complex regulation of *SCARB1*/SR-BI transcription/expression levels.

## Signaling pathways regulated by SR-BI and HDL

**Figure 1:**
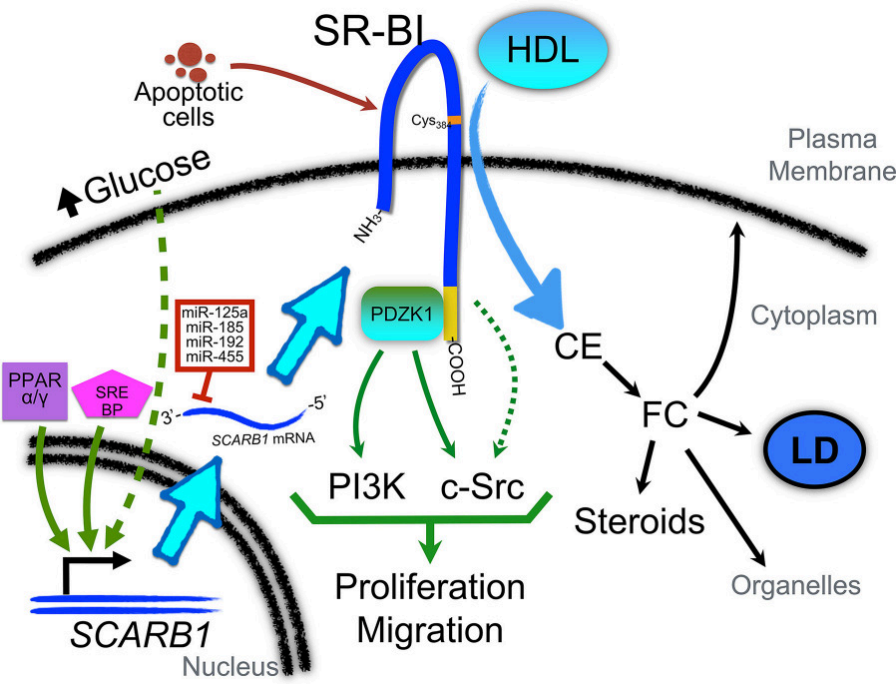
**Role of SR-BI in the regulation of cellular signaling pathways and regulation of its expression in cancer cells**. PPARα and/or PPARγ and SREBP pathways may be activated to promote *SCARB1* gene expression. In addition, as described in macrophages, high levels of glucose may also contribute to SR-BI up-regulation via pathways that remain to be identified. Several miRNAs (such as miR-192, -185, -125a, and -455) have been found to regulate the post-transcriptional expression of SR-BI. Interestingly, these miRs are down-regulated in several cancers. Once associated with the plasma membrane, SR-BI contributes to the uptake of cholesteryl ester (CE) from HDL. A relevant residue in SR-BI for this interaction is Cys384 (denoted with an orange label). CE may be converted into free cholesterol (FC) which can be incorporated in the plasma membrane or organelles (such as mitochondria), stored in lipid droplets (LD), or used as substrate for steroids and other cholesterol metabolites. The C-terminal domain of SR-BI (denoted in yellow) interacts with PDZK1, which can activate PI3-kinase (PI3K) and c-Src pathways to promote proliferation and migration. Another yet unexplored role of SR-BI is its interaction with apoptotic cells. This interaction may also contribute to the activation of signaling pathways that promote malignant features.

Few data are currently available concerning the signaling pathways activated or repressed by SR-BI in cancer cells. We have previously reported that HDL/SR-BI interaction can activate both PI3K and Erk1/2 pathways in two breast cancer cell lines (Danilo et al., 2013). These findings are consistent with results obtained with endothelial cells (Kimura et al., 2010). Besides, SR-BI also interacts with PDZK1, and this interaction is required to mediate eNOS activation and endothelial cell migration. Interestingly, PDZK1 is not required for HDL binding, SR-BI plasma membrane localization, nor cholesteryl ester uptake (Zhu et al., 2008). It has been proposed that PDZK1 is important for HDL/SR-BI-mediated activation of the PI3K (Kimura et al., 2006) and c-Src (Zhu et al., 2008) pathways, and stimulation of migration in endothelial cells (Zhu et al., 2008; Figure 1). Importantly, activation of this signaling cascade has been shown to require the interaction of c-Src and SR-BI cytoplasmic C-terminal domain (Seetharam et al., 2006; Zhu et al., 2008).

Signaling pathways activated by another member of the class B scavenger receptor, CD36, have also been investigated. The C-terminal domain of CD36 has been shown to also physically interact with Src family non-receptor tyrosine kinases (Moore et al., 2002; Rahaman et al., 2006; Tao et al., 2015) that activates a cascade of signaling molecules such as MAPK, p38, JNK, FAK, and GTPases (reviewed in Park, 2014). Conversely, over-expression of a mutant version of SR-BI (lacking the C-terminal residues 465 to 509) has been shown to prevent HDL-induced proliferation in the MCF-7 breast cancer cell line (Cao et al., 2004). Since MCF-7 endogenously express significant levels of SR-BI (Danilo et al., 2013), the later finding implies a competitive effect for the SR-BI mutant in binding HDL and regulating down-stream signaling pathways. Taken together, these data suggest a relevant role for the C-terminal domain of SR-BI in cell proliferation of breast cancer cells.

## SR-BI, regulation of cellular cholesterol homeostasis and cancer

The role of cholesterol in tumor progression has been extensively studied in the past decade. Cholesterol has been shown to regulate essential signaling pathways involved in cellular proliferation, migration, survival, thereby promoting cancer progression. In that regard, several studies have shown that plasma membrane cholesterol plays an important role in these pathways (Zhuang et al., 2005). Furthermore, SR-BI has been shown to regulate cellular cholesterol homeostasis (Phillips, 2014). While CD36 localization in cholesterol-rich plasma membrane and interaction with caveolin-1 has clearly been established (Frank et al., 2002; Ring et al., 2006; Thorne et al., 2006), contradictory results have been obtained concerning SR-BI and its interaction with specific plasma membrane domains and cholesterol. Initial studies by Babitt et al. (1997) have suggested that SR-BI colocalizes with caveolae and is fatty acylated in the CHO-ldla7-mSR-BI and Y1-BS1 cell lines. These findings were confirmed by us using COS-7 (Frank et al., 2002) but Rhainds et al. found SR-BI in lipid raft membrane domains devoid of caveolin-1 in HepG2 cells (Rhainds et al., 2004). However, other studies using WI38[SR-BI] and ACTH-treated murine adrenal Y1-BS1 cells showed that SR-BI was preferentially associated with non-detergent-resistant membranes and no interaction with caveolin-1 was needed to reach the plasma membrane (Peng et al., 2004). Specific culture conditions or cell types may explain some of the observed contradictory results. Nevertheless, the C-terminal transmembrane domain of SR-BI has been shown to interact with cholesterol, and this domain may act as a cholesterol-sensing domain, which function remains to be established (Assanasen et al., 2005). While we have shown that the downregulation of SR-BI in breast cancer cell lines is associated with reduced cellular cholesterol content and reduced tumor aggressivity (Danilo et al., 2013), it remains to be determined whether SR-BI-regulated signaling pathways and cholesterol homeostasis can be uncoupled.

## Role of SR-BI in cancer

Although no study examining the role for SR-BI in the promotion of cell transformation has been performed, it has been recognized that cancer cells can use the HDL/SR-BI pathway to take up cholesteryl ester and enhance malignant phenotypes (Figure 1; Danilo et al., 2013; Zheng et al., 2013). As shown in Table 1, SR-BI expression is up-regulated in Leydig cell tumors, nasopharyngeal carcinoma, and breast and prostate cancers. Among these cancers, the role of HDL/SR-BI in breast and prostate cancers have been the most extensively studied. The breast cancer cell line HBL-100 can take up cholesteryl ester from apoE-depleted HDL_3_ via SR-BI (Pussinen et al., 2000). Additionally, over-expression of SR-BI can enhance HDL-mediated proliferation of the breast cancer cell line MCF-7 via the PI3K/AP-1 pathway (Cao et al., 2004). In further support of a role for HDL/SR-BI in breast cancer growth, we have also demonstrated that HDL/SR-BI interaction is required for breast cancer cells to proliferate and migrate *in vitro*, and SR-BI promotes the growth of tumors *in vivo* (Danilo et al., 2013). Similarly, proliferation of mesenchymal stem cells is also enhanced by HDL/SR-BI interaction where MAPK/ERK1/2 and PI3K/AKT pathways have been inplicated (Xu et al., 2012). Conversely, down-regulation of SR-BI in prostate cancer cells (C4-2 and LNCap cell lines) has been shown to cause a significant reduction in cellular viability and PSA secretion (Twiddy et al., 2012) and inhibit cellular motility in a wound-healing assay of nasopharyngeal cancer cell lines (Zheng et al., 2013). However, studies by Sekine et al. have shown that SR-BI did not regulate HDL-mediated proliferation of the PC-3 prostate cancer cell line (Sekine et al., 2010). Nevertheless, down-regulation of SR-BI has also been observed in adrenocortical carcinomas and testicular seminoma (Table 1). The reasons for these differences are not clear but may involve different requirements for cholesterol uptake by the tumors. In the later cases, cholesterol levels may not be a rate-limiting factor for tumor growth. Alternatively, other isoforms of SR-BI or alternative cholesterol uptake pathways may be upregulated and allow the maintenance of a “tumor specific cholesterol homeostasis.”

While SR-BI mediates HDL-CE uptake in hepatocytes, in peripheral tissues, SR-BI binding to HDL can stimulate a net exit of cholesterol via cholesterol efflux from intracellular stores. In cancer, efflux and influx might be finely regulated to ensure the maintenance of high levels of intracellular cholesterol within proliferative cancer cells. The direction of cholesterol flux may depend on the specific and respective properties of cholesterol acceptors and cellular plasma membrane. Cholesterol efflux mediated by SR-BI highly correlates with HDL phospholipid content present in serum (Fournier et al., 1996, 1997). To further test the role of phospholipid, reconstituted HDL enriched with phosphatidylcholine or sphingomyelin have been used to study efflux and influx mediated by SR-BI in CHO cells (Yancey et al., 2000). It was concluded that sphyngomyelin-enriched HDL enhanced net efflux by decreasing SR-BI-mediated cholesterol influx, whereas phosphatidylcholine enrichment of HDL increased SR-BI-mediated efflux (Yancey et al., 2000). However, it is currently unknown if the same mechanisms can regulate cholesterol homeostasis in cancer cells.

When the concentration of glucose is abnormally elevated as in diabetes, cholesterol metabolism in macrophages is impaired (Hayek et al., 2007; Zhou et al., 2008). Interestingly, a glucose-concentration-dependent increase in SR-BI mRNA and protein levels has been proposed as an explanation for the abnormal cholesterol metabolism observed in macrophages and other cells (Tu and Albers, 2001). Moreover, elevated glucose concentrations can reduce HDL-mediated cholesterol efflux and increase total macrophage cholesterol levels (Gantman et al., 2010). Importantly, glucose uptake is remarkably enhanced in tumors (Cantor and Sabatini, 2012), and previous studies have demonstrated a pro-proliferative role of glucose in the regulation of cellular proliferation of cancer cells (Masur et al., 2011). Since glucose and lipid metabolism are interconnected (reviewed in (Dang, 2012)), these findings suggest that tumor cell exposure to glucose may contribute in cancer cells to SR-BI upregulation that may participate in cancer progression (Figure 1). Interestingly, HDL has also been shown to regulate glucose metabolism in various cell types (Siebel et al., 2015).

## Development of SR-BI inhibitors

SR-BI effect on cholesterol transport has been extensively studied in cardiovascular diseases. Not surprisingly, pharmacological drugs that block SR-BI-mediated HDL-CE uptake have been developed in this particular context. Strategies have been applied to design new specific compounds to target SR-BI. Using high-throughput screening (HTS), BLT-1 (block lipid transport 1) was found to specifically inhibit SR-BI lipid transfer (Nieland et al., 2002). This inhibitor blocks entry of HDL-CE by targeting Cys384 in the extracellular domain of SR-BI (Yu et al., 2011). Importantly, our group has reported that BLT-1 can inhibit SR-BI-mediated cellular proliferation in breast cancer cells (Danilo et al., 2013). Another small molecule, ITX5061, has been shown to increase plasma HDL levels in wild type and human apoA-I transgenic mice in an SR-BI-dependent manner (Masson et al., 2009). Around a decade after BLT-1 discovery, new molecules (ML278, ML279, ML312) were identified by HTS as potent inhibitors with less deleterious effects than ITX5061 and BLT-1 (Faloon et al., 2010a,b,c). More recently, using structural comparative analysis to ITX-5061, a non-absorbable potent compound inhibiting SR-BI has been identified (Sparks et al., 2016).

Antibodies against the extracellular domain of SR-BI have also been developed to prevent HDL binding. Accordingly, a neutralizing antibody against aminoacids 174–356 of murine SR-BI can block both HDL binding and selective cholesteryl ester uptake in murine macrophages (Chen et al., 2000). These new compounds should bring scientists new tools to investigate the role of SR-BI in cancer development and permit the identification of new strategies to inhibit tumor growth and cancer progression.

Recent works have also demonstrated that mimetics of HDL (a.k.a., nanoparticles) can target cells expressing SR-BI. In these cases, studies have shown that tumor cells overexpressing SR-BI could be targeted by nanocomplexes, which may promote the transfer of lipoprotein-derived cargo content (Zheng et al., 2013; McMahon et al., 2015; Julovi et al., 2016). While this approach appears to be efficient, it is important to note that SR-BI is also present at the surface of important non-tumor cells (e.g., hepatocytes, endothelial cells…). Therefore, targeting SR-BI at the surface of tumor cells will require the development of new methods allowing the specific targeting of these cell types.

## Conclusions

Additional studies remain to be performed to better establish the role of SR-BI in cancer. In particular, the role of the different sub-populations of HDL and of other lipoproteins such as LDL needs to be better characterized. Moreover, it remains to be determined whether SR-BI can regulate concommitantly cellular cholesterol homeostasis and various cellular signaling pathways. Therefore, the development of new types of SR-BI inhibitors and the identification of their mechanism of action should provide new information. Alternatively, the use of SR-BI mutants and/or SR-BI/CD36 chimeric constructs should also allow us to better understand the role of SR-BI in cancer.

## Author contributions

All authors contributed significantly to this review. PF and JG design, wrote and review the manuscript. CB and SC contributed to the discussion and correction of the manuscript.

### Conflict of interest statement

The authors declare that the research was conducted in the absence of any commercial or financial relationships that could be construed as a potential conflict of interest.

## References

[B1] Acton S. L. Scherer P. E. Lodish H. F. Krieger M. (1994). Expression cloning of SR-BI, a CD36-related class B scavenger receptor. J. Biol. Chem. 269, 21003–21009. 7520436

[B2] Arenas M. I. Lobo M. V. Caso E. Huerta L. Paniagua R. Martín-Hidalgo M. A. (2004). Normal and pathological human testes express hormone-sensitive lipase and the lipid receptors CLA-1/SR-BI and CD36. Hum. Pathol. 35, 34–42. 10.1016/j.humpath.2003.08.015 14745722

[B3] Assanasen C. Mineo C. Seetharam D. Yuhanna I. S. Marcel Y. L. Connelly M. A.. (2005). Cholesterol binding, efflux, and a PDZ-interacting domain of scavenger receptor-BI mediate HDL-initiated signaling. J. Clin. Invest. 115, 969–977. 10.1172/JCI23858 15841181 PMC1069105

[B4] Azhar S. Leers-Sucheta S. Reaven E. (2003). Cholesterol uptake in adrenal and gonadal tissues: the SR-BI and ‘selective’ pathway connection. Front. Biosci. 8, s998–s1029. 10.2741/1165 12957864

[B5] Babitt J. Trigatti B. Rigotti A. Smart E. J. Anderson R. G. Xu S.. (1997). Murine SR-BI, a high density lipoprotein receptor that mediates selective lipid uptake, is N-glycosylated and fatty acylated and colocalizes with plasma membrane caveolae. J. Biol. Chem. 272, 13242–13249. 10.1074/jbc.272.20.13242 9148942

[B6] Calvo D. Dopazo J. Vega M. A. (1995). The CD36, CLA-1 (CD36L1), and LIMPII (CD36L2) gene family: cellular distribution, chromosomal location, and genetic evolution. Genomics 25, 100–106. 10.1016/0888-7543(95)80114-2 7539776

[B7] Calvo D. Gómez-Coronado D. Lasunción M. A. Vega M. A. (1997). CLA-1 is an 85-kD plasma membrane glycoprotein that acts as a high-affinity receptor for both native (HDL, LDL, and VLDL) and modified (OxLDL and AcLDL) lipoproteins. Arterioscler. Thromb. Vasc. Biol. 17, 2341–2349. 10.1161/01.ATV.17.11.2341 9409200

[B8] Calvo D. Vega M. A. (1993). Identification, primary structure, and distribution of CLA-1, a novel member of the CD36/LIMPII gene family. J. Biol. Chem. 268, 18929–18935. 7689561

[B9] Cantor J. R. Sabatini D. M. (2012). Cancer cell metabolism: one hallmark, many faces. Cancer Discov. 2, 881–898. 10.1158/2159-8290.CD-12-0345 23009760 PMC3491070

[B10] Cao G. Garcia C. K. Wyne K. L. Schultz R. A. Parker K. L. Hobbs H. H. (1997). Structure and localization of the human gene encoding SR-BI/CLA-1. Evidence for transcriptional control by steroidogenic factor 1. J. Biol. Chem. 272, 33068–33076. 10.1074/jbc.272.52.33068 9407090

[B11] Cao W. M. Murao K. Imachi H. Yu X. Abe H. Yamauchi A.. (2004). A mutant high-density lipoprotein receptor inhibits proliferation of human breast cancer cells. Cancer Res. 64, 1515–1521. 10.1158/0008-5472.CAN-03-0675 14973113

[B12] Carithers L. J. Ardlie K. Barcus M. Branton P. A. Britton A. Buia S. A.. (2015). A novel approach to high-quality postmortem tissue procurement: the GTEx project. Biopreserv. Biobank. 13, 311–319. 10.1089/bio.2015.0032 26484571 PMC4675181

[B13] Chai J. Wang S. Han D. Dong W. Xie C. Guo H. (2015). MicroRNA-455 inhibits proliferation and invasion of colorectal cancer by targeting RAF proto-oncogene serine/threonine-protein kinase. Tumour Biol. 36, 1313–1321. 10.1007/s13277-014-2766-3 25355599

[B14] Chen W. Silver D. L. Smith J. D. Tall A. R. (2000). Scavenger receptor-BI inhibits ATP-binding cassette transporter 1- mediated cholesterol efflux in macrophages. J. Biol. Chem. 275, 30794–30800. 10.1074/jbc.M004552200 10896940

[B15] Chinetti G. Gbaguidi F. G. Griglio S. Mallat Z. Antonucci M. Poulain P.. (2000). CLA-1/SR-BI is expressed in atherosclerotic lesion macrophages and regulated by activators of peroxisome proliferator-activated receptors. Circulation 101, 2411–2417. 10.1161/01.CIR.101.20.2411 10821819

[B16] Dang C. V. (2012). Links between metabolism and cancer. Genes Dev. 26, 877–890. 10.1101/gad.189365.112 22549953 PMC3347786

[B17] Danilo C. Frank P. G. (2012). Cholesterol and breast cancer development. Curr. Opin. Pharmacol. 12, 677–682. 10.1016/j.coph.2012.07.009 22867847

[B18] Danilo C. Gutierrez-Pajares J. L. Mainieri M. A. Mercier I. Lisanti M. P. Frank P. G. (2013). Scavenger receptor class B type I regulates cellular cholesterol metabolism and cell signaling associated with breast cancer development. Breast Cancer Res. 15:R87. 10.1186/bcr3483 24060386 PMC3978612

[B19] de Gonzalo-Calvo D. López-Vilaró L. Nasarre L. Perez-Olabarria M. Vazquez T. Escuin D.. (2015). Intratumor cholesteryl ester accumulation is associated with human breast cancer proliferation and aggressive potential: a molecular and clinicopathological study. BMC Cancer 15:460. 10.1186/s12885-015-1469-5 26055977 PMC4460760

[B20] Faloon P. W. Dockendorff C. Germain A. Yu M. Nag P. P. Youngsaye W. (2010b). A Small Molecule Inhibitor of Scavenger Receptor BI-Mediated Lipid Uptake-Probe 2. Probe Reports from the NIH Molecular Libraries Program. Bethesda (MD).

[B21] Faloon P. W. Dockendorff C. Youngsaye W. Yu M. Nag P. P. Lewis T. A. (2010a). A Small Molecule Inhibitor of Scavenger Receptor BI-Mediated Lipid Uptake-Probe 1. Probe Reports from the NIH Molecular Libraries Program. Bethesda (MD).

[B22] Faloon P. W. Dockendorff C. Yu M. Bennion M. Johnston S. Negri J. (2010c). A Small Molecule Inhibitor of Scavenger Receptor BI-Mediated Lipid Uptake-Probe 3. Probe Reports from the NIH Molecular Libraries Program. Bethesda (MD). 23762924

[B23] Feng S. Cong S. Zhang X. Bao X. Wang W. Li H.. (2011). MicroRNA-192 targeting retinoblastoma 1 inhibits cell proliferation and induces cell apoptosis in lung cancer cells. Nucleic Acids Res. 39, 6669–6678. 10.1093/nar/gkr232 21511813 PMC3159440

[B24] Fournier N. de la Llera Moya M. Burkey B. F. Swaney J. B. Paterniti J. Jr. Moatti N.. (1996). Role of HDL phospholipid in efflux of cell cholesterol to whole serum: studies with human apoA-I transgenic rats. J. Lipid Res. 37, 1704–1711. 8864954

[B25] Fournier N. Paul J. L. Atger V. Cogny A. Soni T. de la Llera-Moya M.. (1997). HDL phospholipid content and composition as a major factor determining cholesterol efflux capacity from Fu5AH cells to human serum. Arterioscler. Thromb. Vasc. Biol. 17, 2685–2691. 10.1161/01.ATV.17.11.2685 9409243

[B26] Frank P. G. Marcel Y. L. Connelly M. A. Lublin D. M. Franklin V. Williams D. L.. (2002). Stabilization of caveolin-1 by cellular cholesterol and scavenger receptor class B type I. Biochemistry 41, 11931–11940. 10.1021/bi0257078 12269838

[B27] Freeman M. R. Solomon K. R. (2004). Cholesterol and prostate cancer. J. Cell. Biochem. 91, 54–69. 10.1002/jcb.10724 14689582

[B28] Fu P. Du F. Yao M. Lv K. Liu Y. (2014). MicroRNA-185 inhibits proliferation by targeting c-Met in human breast cancer cells. Exp. Ther. Med. 8, 1879–1883. 10.3892/etm.2014.1999 25371748 PMC4217781

[B29] Fukata Y. Yu X. Imachi H. Nishiuchi T. Lyu J. Seo K.. (2014). 17beta-Estradiol regulates scavenger receptor class BI gene expression via protein kinase C in vascular endothelial cells. Endocrine 46, 644–650. 10.1007/s12020-013-0134-5 24347243

[B30] Gantman A. Fuhrman B. Aviram M. Hayek T. (2010). High glucose stimulates macrophage SR-BI expression and induces a switch in its activity from cholesterol efflux to cholesterol influx. Biochem. Biophys. Res. Commun. 391, 523–528. 10.1016/j.bbrc.2009.11.091 19941833

[B31] Geng L. Chaudhuri A. Talmon G. Wisecarver J. L. Are C. Brattain M.. (2014). MicroRNA-192 suppresses liver metastasis of colon cancer. Oncogene 33, 5332–5340. 10.1038/onc.2013.478 24213572 PMC4016997

[B32] Guo X. Wu Y. Hartley R. S. (2009). MicroRNA-125a represses cell growth by targeting HuR in breast cancer. RNA Biol. 6, 575–583. 10.4161/rna.6.5.10079 19875930 PMC3645467

[B33] Harder C. J. Meng A. Rippstein P. McBride H. M. McPherson R. (2007). SR-BI undergoes cholesterol-stimulated transcytosis to the bile canaliculus in polarized WIF-B cells. J. Biol. Chem. 282, 1445–1455. 10.1074/jbc.M604627200 17105723

[B34] Hayek T. Kaplan M. Kerry R. Aviram M. (2007). Macrophage NADPH oxidase activation, impaired cholesterol fluxes, and increased cholesterol biosynthesis in diabetic mice: a stimulatory role for D-glucose. Atherosclerosis 195, 277–286. 10.1016/j.atherosclerosis.2006.12.026 17258748

[B35] He H. Xu F. Huang W. Luo S. Y. Lin Y. T. Zhang G. H.. (2015). miR-125a-5p expression is associated with the age of breast cancer patients. Genet. Mol. Res. 14, 17927–17933. 10.4238/2015.December.22.17 26782438

[B36] Hu Z. Shen W. J. Kraemer F. B. Azhar S. (2012). MicroRNAs 125a and 455 repress lipoprotein-supported steroidogenesis by targeting scavenger receptor class B type I in steroidogenic cells. Mol. Cell. Biol. 32, 5035–5045. 10.1128/MCB.01002-12 23045399 PMC3510537

[B37] Huby T. Doucet C. Dachet C. Ouzilleau B. Ueda Y. Afzal V.. (2006). Knockdown expression and hepatic deficiency reveal an atheroprotective role for SR-BI in liver and peripheral tissues. J. Clin. Invest. 116, 2767–2776. 10.1172/JCI26893 16964311 PMC1560348

[B38] Imachi H. Murao K. Sayo Y. Hosokawa H. Sato M. Niimi M.. (1999). Evidence for a potential role for HDL as an important source of cholesterol in human adrenocortical tumors via the CLA-1 pathway. Endocr. J. 46, 27–34. 10.1507/endocrj.46.27 10426565

[B39] Julovi S. M. Xue A. Thanh L. T. Gill A. J. Bulanadi J. C. Patel M.. (2016). Apolipoprotein A-II plus lipid emulsion enhance cell growth via SR-B1 and target pancreatic cancer *in vitro* and *in vivo*. PLoS ONE 11:e0151475. 10.1371/journal.pone.0151475 27002321 PMC4803224

[B40] Kim H. Abd Elmageed Z. Y. Davis C. El-Bahrawy A. H. Naura A. S. Ekaidi I.. (2014). Correlation between PDZK1, Cdc37, Akt and breast cancer malignancy: the role of PDZK1 in cell growth through Akt stabilization by increasing and interacting with Cdc37. Mol. Med. 20, 270–279. 10.2119/molmed.2013.00166 24869908 PMC4107102

[B41] Kimura T. Tomura H. Mogi C. Kuwabara A. Damirin A. Ishizuka T.. (2006). Role of scavenger receptor class B type I and sphingosine 1-phosphate receptors in high density lipoprotein-induced inhibition of adhesion molecule expression in endothelial cells. J. Biol. Chem. 281, 37457–37467. 10.1074/jbc.M605823200 17046831

[B42] Kimura T. Tomura H. Sato K. Ito M. Matsuoka I. Im D. S.. (2010). Mechanism and role of high density lipoprotein-induced activation of AMP-activated protein kinase in endothelial cells. J. Biol. Chem. 285, 4387–4397. 10.1074/jbc.M109.043869 20018878 PMC2836043

[B43] Kocher O. Birrane G. Tsukamoto K. Fenske S. Yesilaltay A. Pal R.. (2010). *In vitro* and *in vivo* analysis of the binding of the C terminus of the HDL receptor scavenger receptor class B, type I (SR-BI), to the PDZ1 domain of its adaptor protein PDZK1. J. Biol. Chem. 285, 34999–35010. 10.1074/jbc.M110.164418 20739281 PMC2966114

[B44] Kocher O. Yesilaltay A. Cirovic C. Pal R. Rigotti A. Krieger M. (2003). Targeted disruption of the PDZK1 gene in mice causes tissue-specific depletion of the high density lipoprotein receptor scavenger receptor class B type I and altered lipoprotein metabolism. J. Biol. Chem. 278, 52820–52825. 10.1074/jbc.M310482200 14551195

[B45] Krycer J. R. Brown A. J. (2013). Cholesterol accumulation in prostate cancer: a classic observation from a modern perspective. Biochim. Biophys. Acta 1835, 219–229. 10.1016/j.bbcan.2013.01.002 23357067

[B46] Kuzu O. F. Noory M. A. Robertson G. P. (2016). The role of cholesterol in cancer. Cancer Res. 76, 2063–2070. 10.1158/0008-5472.CAN-15-2613 27197250 PMC5813477

[B47] Lee M. Kim E. J. Jeon M. J. (2016). MicroRNAs 125a and 125b inhibit ovarian cancer cells through post-transcriptional inactivation of EIF4EBP1. Oncotarget 7, 8726–8742. 10.18632/oncotarget.6474 26646586 PMC4891000

[B48] Li S. Ma Y. Hou X. Liu Y. Li K. Xu S.. (2015). MiR-185 acts as a tumor suppressor by targeting AKT1 in non-small cell lung cancer cells. Int. J. Clin. Exp. Pathol. 8, 11854–11862. 26617940 PMC4637756

[B49] Li X. Peegel H. Menon K. M. (1998). *In situ* hybridization of high density lipoprotein (scavenger, type 1) receptor messenger ribonucleic acid (mRNA) during folliculogenesis and luteinization: evidence for mRNA expression and induction by human chorionic gonadotropin specifically in cell types that use cholesterol for steroidogenesis. Endocrinology 139, 3043–3049. 10.1210/en.139.7.3043 9645674

[B50] Li Y. J. Ping C. Tang J. Zhang W. (2016). MicroRNA-455 suppresses non-small cell lung cancer through targeting ZEB1. Cell Biol. Int. 40, 621–628. 10.1002/cbin.10584 26801503

[B51] Liu J. Voutilainen R. Heikkila P. Kahri A. I. (1997). Ribonucleic acid expression of the CLA-1 gene, a human homolog to mouse high density lipoprotein receptor SR-BI, in human adrenal tumors and cultured adrenal cells. J. Clin. Endocrinol. Metab. 82, 2522–2527. 10.1210/jc.82.8.2522 9253328

[B52] Llaverias G. Danilo C. Mercier I. Daumer K. Capozza F. Williams T. M.. (2011). Role of cholesterol in the development and progression of breast cancer. Am. J. Pathol. 178, 402–412. 10.1016/j.ajpath.2010.11.005 21224077 PMC3069824

[B53] Llaverias G. Danilo C. Wang Y. Witkiewicz A. K. Daumer K. Lisanti M. P.. (2010). A Western-type diet accelerates tumor progression in an autochthonous mouse model of prostate cancer. Am. J. Pathol. 177, 3180–3191. 10.2353/ajpath.2010.100568 21088217 PMC2993263

[B54] Malerød L. Juvet L. K. Hanssen-Bauer A. Eskild W. Berg T. (2002). Oxysterol-activated LXRalpha/RXR induces hSR-BI-promoter activity in hepatoma cells and preadipocytes. Biochem. Biophys. Res. Commun. 299, 916–923. 10.1016/S0006-291X(02)02760-2 12470667

[B55] Masson D. Koseki M. Ishibashi M. Larson C. J. Miller S. G. King B. D.. (2009). Increased HDL cholesterol and apoA-I in humans and mice treated with a novel SR-BI inhibitor. Arterioscler. Thromb. Vasc. Biol. 29, 2054–2060. 10.1161/ATVBAHA.109.191320 19815817 PMC2783626

[B56] Masur K. Vetter C. Hinz A. Tomas N. Henrich H. Niggemann B.. (2011). Diabetogenic glucose and insulin concentrations modulate transcriptome and protein levels involved in tumour cell migration, adhesion and proliferation. Br. J. Cancer 104, 345–352. 10.1038/sj.bjc.6606050 21179032 PMC3031898

[B57] McMahon K. M. Foit L. Angeloni N. L. Giles F. J. Gordon L. I. Thaxton C. S. (2015). Synthetic high-density lipoprotein-like nanoparticles as cancer therapy. Cancer Treat. Res. 166, 129–150. 10.1007/978-3-319-16555-4_6 25895867 PMC4418545

[B58] Moore K. J. El Khoury J. Medeiros L. A. Terada K. Geula C. Luster A. D.. (2002). A CD36-initiated signaling cascade mediates inflammatory effects of beta-amyloid. J. Biol. Chem. 277, 47373–47379. 10.1074/jbc.M208788200 12239221

[B59] Murai T. (2015). Cholesterol lowering: role in cancer prevention and treatment. Biol. Chem. 396, 1–11. 10.1515/hsz-2014-0194 25205720

[B60] Mysore R. Zhou Y. Sadevirta S. Savolainen-Peltonen H. Nidhina Haridas P. A. Soronen J.. (2016). MicroRNA-192^*^ impairs adipocyte triglyceride storage. Biochim. Biophys. Acta 1864, 342–351. 10.1016/j.bbalip.2015.12.019 26747651

[B61] Nakamura T. Shibata N. Nishimoto-Shibata T. Feng D. Ikemoto M. Motojima K.. (2005). Regulation of SR-BI protein levels by phosphorylation of its associated protein, PDZK1. Proc. Natl. Acad. Sci. U.S.A. 102, 13404–13409. 10.1073/pnas.0506679102 16174736 PMC1224665

[B62] Nieland T. J. Penman M. Dori L. Krieger M. Kirchhausen T. (2002). Discovery of chemical inhibitors of the selective transfer of lipids mediated by the HDL receptor SR-BI. Proc. Natl. Acad. Sci. U.S.A. 99, 15422–15427. 10.1073/pnas.222421399 12438696 PMC137732

[B63] Norata G. D. Ongari M. Uboldi P. Pellegatta F. Catapano A. L. (2005). Liver X receptor and retinoic X receptor agonists modulate the expression of genes involved in lipid metabolism in human endothelial cells. Int. J. Mol. Med. 16, 717–722. 10.3892/ijmm.16.4.717 16142410

[B64] Park Y. M. (2014). CD36, a scavenger receptor implicated in atherosclerosis. Exp. Mol. Med. 46:e99. 10.1038/emm.2014.38 24903227 PMC4081553

[B65] Pello O. M. De Pizzol M. Mirolo M. Soucek L. Zammataro L. Amabile A.. (2012). Role of c-MYC in alternative activation of human macrophages and tumor-associated macrophage biology. Blood 119, 411–421. 10.1182/blood-2011-02-339911 22067385

[B66] Peng Y. Akmentin W. Connelly M. A. Lund-Katz S. Phillips M. C. Williams D. L. (2004). Scavenger receptor BI (SR-BI) clustered on microvillar extensions suggests that this plasma membrane domain is a way station for cholesterol trafficking between cells and high-density lipoprotein. Mol. Biol. Cell 15, 384–396. 10.1091/mbc.E03-06-0445 14528013 PMC307555

[B67] Phillips M. C. (2014). Molecular mechanisms of cellular cholesterol efflux. J. Biol. Chem. 289, 24020–24029. 10.1074/jbc.R114.583658 25074931 PMC4148835

[B68] Pussinen P. J. Karten B. Wintersperger A. Reicher H. McLean M. Malle E.. (2000). The human breast carcinoma cell line HBL-100 acquires exogenous cholesterol from high-density lipoprotein via CLA-1 (CD-36 and LIMPII analogous 1)-mediated selective cholesteryl ester uptake. Biochem. J. 349(Pt 2), 559–566. 10.1042/bj3490559 10880355 PMC1221179

[B69] Qu F. Cui X. Hong Y. Wang J. Li Y. Chen L.. (2013). MicroRNA-185 suppresses proliferation, invasion, migration, and tumorigenicity of human prostate cancer cells through targeting androgen receptor. Mol. Cell. Biochem. 377, 121–130. 10.1007/s11010-013-1576-z 23417242

[B70] Rahaman S. O. Lennon D. J. Febbraio M. Podrez E. A. Hazen S. L. Silverstein R. L. (2006). A CD36-dependent signaling cascade is necessary for macrophage foam cell formation. Cell Metab. 4, 211–221. 10.1016/j.cmet.2006.06.007 16950138 PMC1855263

[B71] Rhainds D. Bourgeois P. Bourret G. Huard K. Falstrault L. Brissette L. (2004). Localization and regulation of SR-BI in membrane rafts of HepG2 cells. J. Cell Sci. 117(Pt 15), 3095–3105. 10.1242/jcs.01182 15226391

[B72] Ring A. Le Lay S. Pohl J. Verkade P. Stremmel W. (2006). Caveolin-1 is required for fatty acid translocase (FAT/CD36) localization and function at the plasma membrane of mouse embryonic fibroblasts. Biochim. Biophys. Acta 1761, 416–423. 10.1016/j.bbalip.2006.03.016 16702023

[B73] Schörghofer D. Kinslechner K. Preitschopf A. Schütz B. Röhrl C. Hengstschlager M.. (2015). The HDL receptor SR-BI is associated with human prostate cancer progression and plays a possible role in establishing androgen independence. Reprod. Biol. Endocrinol. 13:88. 10.1186/s12958-015-0087-z 26251134 PMC4528807

[B74] Seetharam D. Mineo C. Gormley A. K. Gibson L. L. Vongpatanasin W. Chambliss K. L.. (2006). High-density lipoprotein promotes endothelial cell migration and reendothelialization via scavenger receptor-B type I. Circ. Res. 98, 63–72. 10.1161/01.RES.0000199272.59432.5b 16339487

[B75] Sekine Y. Demosky S. J. Stonik J. A. Furuya Y. Koike H. Suzuki K.. (2010). High-density lipoprotein induces proliferation and migration of human prostate androgen-independent cancer cells by an ABCA1-dependent mechanism. Mol. Cancer Res. 8, 1284–1294. 10.1158/1541-7786.MCR-10-0008 20671065 PMC2941551

[B76] Siebel A. L. Heywood S. E. Kingwell B. A. (2015). HDL and glucose metabolism: current evidence and therapeutic potential. Front. Pharmacol. 6:258. 10.3389/fphar.2015.00258 26582989 PMC4628107

[B77] Silvente-Poirot S. Poirot M. (2012). Cholesterol metabolism and cancer: the good, the bad and the ugly. Curr. Opin. Pharmacol. 12, 673–676. 10.1016/j.coph.2012.10.004 23103112

[B78] Silver D. L. (2002). A carboxyl-terminal PDZ-interacting domain of scavenger receptor B, type I is essential for cell surface expression in liver. J. Biol. Chem. 277, 34042–34047. 10.1074/jbc.M206584200 12119305

[B79] Silver D. L. Jiang X. C. Arai T. Bruce C. Tall A. R. (2000). Receptors and lipid transfer proteins in HDL metabolism. Ann. N. Y. Acad. Sci. 902, 103–111; discussion 11–2. 10.1111/j.1749-6632.2000.tb06305.x 10865830

[B80] Simko V. Ginter E. (2014). Understanding cholesterol: high is bad but too low may also be risky – is low cholesterol associated with cancer? Bratisl. Lek. Listy 115, 59–65. 10.4149/bll_2014_013 24601696

[B81] Sparks S. M. Zhou H. Generaux C. Harston L. Moncol D. Jayawickreme C.. (2016). Identification of nonabsorbable inhibitors of the scavenger receptor-BI (SR-BI) for tissue-specific administration. Bioorg. Med. Chem. Lett. 26, 1901–1904. 10.1016/j.bmcl.2016.03.025 26988301

[B82] Sun J. Fan Z. Lu S. Yang J. Hao T. Huo Q. (2016). miR-192 suppresses the tumorigenicity of prostate cancer cells by targeting and inhibiting nin one binding protein. Int. J. Mol. Med. 37, 485–492. 10.3892/ijmm.2016.2449 26743688

[B83] Tang H. Liu P. Yang L. Xie X. Ye F. Wu M.. (2014). miR-185 suppresses tumor proliferation by directly targeting E2F6 and DNMT1 and indirectly upregulating BRCA1 in triple-negative breast cancer. Mol. Cancer Ther. 13, 3185–3197. 10.1158/1535-7163.MCT-14-0243 25319390

[B84] Tao H. Yancey P. G. Babaev V. R. Blakemore J. L. Zhang Y. Ding L.. (2015). Macrophage SR-BI mediates efferocytosis via Src/PI3K/Rac1 signaling and reduces atherosclerotic lesion necrosis. J. Lipid Res. 56, 1449–1460. 10.1194/jlr.M056689 26059978 PMC4513986

[B85] Thorne R. F. Law E. G. Elith C. A. Ralston K. J. Bates R. C. Burns G. F. (2006). The association between CD36 and Lyn protein tyrosine kinase is mediated by lipid. Biochem. Biophys. Res. Commun. 351, 51–56. 10.1016/j.bbrc.2006.09.156 17052693

[B86] Tong Z. Liu N. Lin L. Guo X. Yang D. Zhang Q. (2015). miR-125a-5p inhibits cell proliferation and induces apoptosis in colon cancer via targeting BCL2, BCL2L12 and MCL1. Biomed. Pharmacother. 75, 129–136. 10.1016/j.biopha.2015.07.036 26297542

[B87] Towns R. Menon K. M. (2005). The role of cyclic AMP response element binding protein in transactivation of scavenger receptor class B type I promoter in transfected cells and in primary cultures of rat theca-interstitial cells. Mol. Cell. Endocrinol. 245, 23–30. 10.1016/j.mce.2005.09.013 16298471

[B88] Tréguier M. Doucet C. Moreau M. Dachet C. Thillet J. Chapman M. J.. (2004). Transcription factor sterol regulatory element binding protein 2 regulates scavenger receptor Cla-1 gene expression. Arterioscler. Thromb. Vasc. Biol. 24, 2358–2364. 10.1161/01.ATV.0000147896.69299.85 15486308

[B89] Trigatti B. Rigotti A. Krieger M. (2000). The role of the high-density lipoprotein receptor SR-BI in cholesterol metabolism. Curr. Opin. Lipidol. 11, 123–131. 10.1097/00041433-200004000-00004 10787173

[B90] Tu A. Y. Albers J. J. (2001). Glucose regulates the transcription of human genes relevant to HDL metabolism: responsive elements for peroxisome proliferator-activated receptor are involved in the regulation of phospholipid transfer protein. Diabetes 50, 1851–1856. 10.2337/diabetes.50.8.1851 11473048

[B91] Twiddy A. L. Cox M. E. Wasan K. M. (2012). Knockdown of scavenger receptor class B type I reduces prostate specific antigen secretion and viability of prostate cancer cells. Prostate 72, 955–965. 10.1002/pros.21499 22025344

[B92] UniProt C. (2015). UniProt: a hub for protein information. Nucleic Acids Res. 43, D204–D212. 10.1093/nar/gku989 25348405 PMC4384041

[B93] Wang L. Jia X. J. Jiang H. J. Du Y. Yang F. Si S. Y.. (2013). MicroRNAs 185, 96, and 223 repress selective high-density lipoprotein cholesterol uptake through posttranscriptional inhibition. Mol. Cell. Biol. 33, 1956–1964. 10.1128/MCB.01580-12 23459944 PMC3647964

[B94] Webb N. R. Connell P. M. Graf G. A. Smart E. J. de Villiers W. J. de Beer F. C.. (1998). SR-BII, an isoform of the scavenger receptor BI containing an alternate cytoplasmic tail, mediates lipid transfer between high density lipoprotein and cells. J. Biol. Chem. 273, 15241–15248. 10.1074/jbc.273.24.15241 9614139

[B95] Webb N. R. de Villiers W. J. Connell P. M. de Beer F. C. van der Westhuyzen D. R. (1997). Alternative forms of the scavenger receptor BI (SR-BI). J. Lipid Res. 38, 1490–1495. 9254074

[B96] Wiersma H. Gatti A. Nijstad N. Kuipers F. Tietge U. J. (2009b). Hepatic SR-BI, not endothelial lipase, expression determines biliary cholesterol secretion in mice. J. Lipid Res. 50, 1571–1580. 10.1194/jlr.M800434-JLR200 19252221 PMC2724056

[B97] Wiersma H. Gatti A. Nijstad N. Oude Elferink R. P. Kuipers F. Tietge U. J. (2009a). Scavenger receptor class B type I mediates biliary cholesterol secretion independent of ATP-binding cassette transporter g5/g8 in mice. Hepatology 50, 1263–1272. 10.1002/hep.23112 19637290

[B98] Wood P. Mulay V. Darabi M. Chan K. C. Heeren J. Pol A.. (2011). Ras/mitogen-activated protein kinase (MAPK) signaling modulates protein stability and cell surface expression of scavenger receptor SR-BI. J. Biol. Chem. 286, 23077–23092. 10.1074/jbc.M111.236398 21525007 PMC3123075

[B99] Xu J. Qian J. Xie X. Lin L. Ma J. Huang Z.. (2012). High density lipoprotein cholesterol promotes the proliferation of bone-derived mesenchymal stem cells via binding scavenger receptor-B type I and activation of PI3K/Akt, MAPK/ERK1/2 pathways. Mol. Cell. Biochem. 371, 55–64. 10.1007/s11010-012-1422-8 22886428

[B100] Yancey P. G. de la Llera-Moya M. Swarnakar S. Monzo P. Klein S. M. Connelly M. A.. (2000). High density lipoprotein phospholipid composition is a major determinant of the bi-directional flux and net movement of cellular free cholesterol mediated by scavenger receptor BI. J. Biol. Chem. 275, 36596–36604. 10.1074/jbc.M006924200 10964930

[B101] Yu M. Romer K. A. Nieland T. J. Xu S. Saenz-Vash V. Penman M.. (2011). Exoplasmic cysteine Cys384 of the HDL receptor SR-BI is critical for its sensitivity to a small-molecule inhibitor and normal lipid transport activity. Proc. Natl. Acad. Sci. U.S.A. 108, 12243–12248. 10.1073/pnas.1109078108 21746906 PMC3145699

[B102] Yuan B. Wu C. Wang X. Wang D. Liu H. Guo L.. (2016). High scavenger receptor class B type I expression is related to tumor aggressiveness and poor prognosis in breast cancer. Tumour Biol. 37, 3581–3588. 10.1007/s13277-015-4141-4 26456958

[B103] Zhang Z. Liu X. Feng B. Liu N. Wu Q. Han Y.. (2015). STIM1, a direct target of microRNA-185, promotes tumor metastasis and is associated with poor prognosis in colorectal cancer. Oncogene 34, 4808–4820. 10.1038/onc.2014.404 25531324 PMC4569941

[B104] Zheng Y. Liu Y. Jin H. Pan S. Qian Y. Huang C.. (2013). Scavenger receptor B1 is a potential biomarker of human nasopharyngeal carcinoma and its growth is inhibited by HDL-mimetic nanoparticles. Theranostics 3, 477–486. 10.7150/thno.6617 23843895 PMC3706691

[B105] Zhou H. Tan K. C. Shiu S. W. Wong Y. (2008). Cellular cholesterol efflux to serum is impaired in diabetic nephropathy. Diabetes Metab. Res. Rev. 24, 617–623. 10.1002/dmrr.895 18802933

[B106] Zhu W. Saddar S. Seetharam D. Chambliss K. L. Longoria C. Silver D. L.. (2008). The scavenger receptor class B type I adaptor protein PDZK1 maintains endothelial monolayer integrity. Circ. Res. 102, 480–487. 10.1161/CIRCRESAHA.107.159079 18174467

[B107] Zhuang L. Kim J. Adam R. M. Solomon K. R. Freeman M. R. (2005). Cholesterol targeting alters lipid raft composition and cell survival in prostate cancer cells and xenografts. J. Clin. Invest. 115, 959–968. 10.1172/JCI200519935 15776112 PMC1064980

